# Antizymotics, or Agents Which Prevent Fermentation and Putrefaction

**Published:** 1892-12

**Authors:** N. S. Hoff


### THE DENTAL REGISTER.

Vol. XLVI.] DECEMBER, 1892. [No. 12.

### Communications.

### Antizymotics, or Agents which Prevent Fermentation and Putrefaction.

BY N. S. HOFF, D.D.S.

Read before the Michigan State Dental Society.

So much dependence is placed upon the efficiency of this class of drugs by the surgical profession that it seems desirable to know all that is possible as to the manner in which they operate when brought into contact with the tissues of the human body, and it seems desirable also that some classification should be made to assist in keeping in the memory the manner in which they should be exhibited to secure definite therapeutical results. It is evident that a classification based on our present knowledge of the cause of fermentation and putrefaction can not be entirely satisfactory, but due consideration should also be given to the results of clinical, though it be empirical, practice. We believe that a classification can be made that will bear out all the accepted evidence of scientific investigation and of clinical practice, and we submit a brief sketch of the principles involved in the destruction of living tissues by fermentation and putrefaction, hoping to make evident the basis of a classification which we think is not only scientific, but which will prove clinically practicable and satisfactory.

From our present knowledge of the action of the various agents producing destruction of organized tissue of both the vegetable and animal nature we are unable to make a differential statement that will be truly scientific. We would like to believe that fermentation was a peculiar process taking place in vegetable substances only, and that putrefaction was only to be found where animal tissue was being broken down through the agencies of fermenting substances or minute organisms. The definition of the terms antizymotic and antiseptic would seem to convey this idea; at least the derivation of the words would imply that such a distinction might be made. Antizymotic is derived from *Anti* —against and *zumosis*—fermentation. Antiseptic is derived from *Anti*—against and *Sepo*—to putrify.

The process of fermentation is one that has a wide range in nature. It is the means by which existing organizations are broken down into molecular conditions, so that growth and development may take place and even life itself be perpetuated. For instance, food taken into the alimentary canal meets first with a substance in the saliva called ptyalin, which changes its starch into a substance that can be appropriated by those absorbent structures whose business it is to secure the pabulum for the proper nutrition of the tissue of the body. Gastric juice and the pancreatic fluid each contain a peculiar ferment which selects its peculiar kind of food and breaks it up into its elements, so that the absorbents may appropriate them to nourish growing tissue cells. We have an excellent illustration in the rotting of grain where a peculiar substance called disastase, has the property of changing the nature of the starch contained in the grain to an entirely different substance, so that the plant that is to grow from the seed may appropriate it for nourishment.

This class of ferments are called organic, because they are the product of an organized body, but they possess no organization, such as we find in another important class of ferments which are organized.

The organic ferments are chemical but not vital substances, and in the economy of nature they are useful agencies in procuring food and nourishment for organized living bodies, but are not largely concerned in the destruction of living tissue in the production of disease.

The organized ferments have a distinct and definite organization. They, under favorable conditions, exhibit all the phenomena of life. They are capable of locomotion, grow and reproduce their kind. They exhibit many phenomena of animal life, yet microscopic investigation has determined their place as belonging to the vegetable kingdom. They are essentially seeds of plants. A body of protoplasm and a cellular wall or covering of a ligneous nature. Some are capable of destroying life in tissue and creating disease, and are called pathogenic; others are harmless or non-pathogenic. Some need air or oxygen to support life, aerobic; others live without air and are anaerobic.

Putrefaction is always accompanied by the presence of bacteria; and bacteriologists maintain that this process can not take place without the presence of micro-organism. It has been reasonably demonstrated that certain micro-organisms are the definite cause of specific diseases. The cholera bacillus has been successfully isolated, and the disease produced in healthy animals by inoculation. This is also true of many others; anthrax, typhoid fever, consumption, etc., can be produced by inoculating healthy animals with pure specimens of the micro-organisms found in these diseases. It is the confident expectation of bacteriologists that characteristic bacteria of every known disease will be determined by further research. Just how these minute organisms produce such destructive changes in living tissue is not definitely known. It has been observed that conditions favorable to their growth are moisture, body temperature, and a favorable medium. Excessive temperature, hot or cold, will inhibit their growth, or destroy them. 105° F will inhibit most varieties; at 32° F they will not grow. They will not grow in either an acid or alkaline medium. It is evident that the process is very closely allied if not identical with fermentation. It has been found that there is present in tissue affected with pathogenic bacteria a peculiar nitrogenous waste product, which has evidently been produced by the activity of the micro-organisms. It very closely resembles vegetable alkaloids, which are formed in putrefying mixture, and is usually poisonous. It is called a ptomain (from *ptoma*—a corpse) because it was first isolated from dead bodies. It is the ptomain that produces in animals the characteristic disease, poisonous or fatal results.

There exist in the body at all times various and numerous species or forms of bacteria. It is a wise provision of nature that it is so, for we find that the presence of the non-pathogenic orders have a decided tendency to modify or correct the action of the disease-producing species. The favorable conditions and presence of germs in so great a variety would seem to promise the speedy overthrow of all vital function in the tissues of the body were it not for the fact that nature has made provision for resisting the encroachment and interference of these organisms, which is brought about principally in three ways.

First, the fluids of the body are capable of destroying them by their acid character, or prevent their growth by their alkaline reaction.

Second, the white corpuscles of the blood and the connective tissue cells have the power to destroy the bacteria by taking them into their interior and digesting them. This can only be accomplished to a certain extent, and when more bacteria are present than the cells can digest, the cells themselves give way and are destroyed by the bacteria, and we have death and the putrefactive process set up.

In the third place, the bacteria overcome and destroy each other or are destroyed by their own products. If a wound is inoculated with several kinds of bacteria, one species will gain the ascendency at the expense of the others, until it has by destruction so contaminated the media in which it is operating by its own activity and has produced a condition in which it can not grow; an excess of acid, or alkali, or alcohol; then its activity ceases and the germ becomes incompetent to produce its species. At this stage another kind of germ that thrives on the conditions present may take up the fight and change conditions to his own hurt or disadvantage, and he in turn is compelled to give way to a more vigorous successor.

This kind of a warfare will continue indefinitely, or, until by surgical and medical interference or renewed and reinforced vital function the system is enabled to overcome the parasitic influence and reestablish a normal and vigorous functional condition.

It is at this point that the interference of the surgeon or physician is needed to turn the process in favor of the attacked structures.

In order that the disinfecting process may be intelligently accomplished it is not necessary that we recognize the peculiar variety of micro-organisms which may be present, as fortunately the larger number are susceptible to, and will succumb to comparatively harmless agencies, and all can be reached by drugs contained in our materia medica.

The conditions produced by the different species are varied, according to the germ, but those which are especially concerned in the formation of gases and odors are of particular interest to the dentist. But all forms are liable to produce both gases and odors under the conditions present in most dental lesions, viz.: putrefaction of the pulp, alveolar abscess, pyorrhœa alveolaris, etc. It is not important here to enumerate the particular action of special species or forms.

We must classify all agents or drugs which may be used to overcome the influence or results of the action of micro-organisms under the general head of antizymotics, that is, agents which will prevent fermentation, for we are not justified in separating fermentation from putrefaction, for fermentation is an essential feature of putrefaction even in animal structures.

In the clinical application of antizymotics for the correction of disease, we find that all drugs and methods to be efficient must be applied in such concentration or power to effect the desired results, that continued application would result in the destruction of large amounts of valuable tissue that is not at all or only slightly affected by the encroachment of zymotic influences. And we have learned also that tissue which is not inoculated or only slightly affected can be kept in an aseptic condition by attenuated solutions of strong germicides or by milder agents, which we will designate antiseptics. We will, therefore, make a sub-classification of antizymotics into disinfectants and antiseptics. The disinfectant is the means whereby we aim to remove all infectious matter and agencies from the tissue; the antiseptic is the means used to preserve the cleanliness obtained by the use of the disinfectant.

Classification:

- Antizymotics.

  - *a* Disinfectants

    - 1 Deodorants.

    - 2 Detergents.

    - 3 Germicides.

  - *b* Antiseptics—no sub-division.

The disinfectant being the first agent applied in the treatment of a diseased condition we will define and discuss it first.

In the examination of a putrid lesion we usually find three conditions prominently present. First, an offensive odor; second, the presence of more or less disorganized and lifeless tissue structure; and third, we conclude from the nature of the process that putrefaction is going on and destructive micro-organisms are present in excess.

We have therefore three apparent and distinct conditions to contend with, and we are compelled to again divide our disinfectant process into three divisions. For our own convenience, reasons that are plainly obvious, we will first aim to overcome the obnoxious smell, and for this purpose we will make use of a *deodorizer.* Second; we will need to remove all the decomposed tissue and such as can not be restored to normal condition. For this purpose detergent measures will be required. Third; all fermenting and putrefactive agencies must be destroyed in order that the tissues may unhindered take on recuperative action. For this purpose germicidal measures will be resorted to. We then have disinfectants divided into deodorants, detergents and germicides, each indicating its purpose and place in therapeutic application.

In the practical application of these agents we find no sharp or distinct lines can be drawn, but the deodorants, detergents and germicides overlap each other, and it is possible to find all three virtues embodied in one agent. But in order to make our subject and classification clear, we will discuss only the virtue of the drug or method which applies to the particular division under consideration.

Deodorizing measures embrace First, the absorption or masking of the odoriferous gases in somewhat of a mechanical way; as by charcoal, lime, earth, or highly volatile drugs such as the essential oils, carbolic acid, etc. These non volatile absorbents are the most durable and permanent in action. Secondly, the union of the gases chemically with agents which change their character and destroy the effluvia. The gases resulting from putrefaction are principally sulphuretted hydrogen, $H_2$ S. nitrogen, and ammonia (N. $H_3$.) And the deodorants are drugs which will give up oxygen or chlorine to unite with these gases to form sulphuric, hydrochloric and nitric acids. These compounds if formed in sufficient quantity and the possibilities of extensive dilution prevented, would act primarily as deodorants, secondly as detergents, as they would burn out the dead tissue, and finally as germicides because they would destroy the contained micro organisms. Thus it is theoretically if not practicably possible to completely disinfect a putrid mass, as for example the pulp of a tooth, by the application of deodorizing agents alone. The most satisfactory results will be had by endeavoring to do only one thing at a time, and although deodorizing may be only temporary in character, it will serve its purpose, and the other stages in disinfection will necessarily follow in regular order and in due time.

The *detergent measures* are more important, and may be divided under two heads, viz: surgical and chemical.

Under surgical measures we would include all operations for the removal of dead or diseased tissue that was beyond hope of restoration to normal function, by instruments, douches, actual cautery, and other mechanical means. These are the most important because, thorough, quick, convenient, safe and definite. It is not necessary here to undertake any description of these processes, as they are all familiarly made use of by dentists in the daily routine of office practice. The excavator, chisel, scaler, lancet, burring engine, and water syringe are detergent measures familiar to every dental surgeon.

The chemical detergents are no less important because of their necessity. But they must be given the second place because, they are slower in action, more painful, and not so thorough nor under such perfect control. Yet they are essential because many of the conditions requiring the use of detergents are so deep seated or so peculiarly environed that, extensive and destructive operations would be required. Operations of great delicacy are sometimes better done by these agents, for instance the removal of a portion of a diseased pulp. Idiosyncrasies of patients will frequently prohibit the use of instruments and resort must be had to chemical agencies; as for example in opening to an alveolar abscess. This class includes the oxidizers, escharotics and cauterants.

*The germicides*, the third division of disinfectants must be such agents or methods as may be applied easily and without causing injury to the structures associated with the disease.

The fact that extremes of heat and cold and all such drugs as are effectively used to destroy micro-organisms in inorganic solutions in the laboratory are injurious to living tissue, makes the proper selection of a medicinal germicide one of considerable importance.

The use of extremes of temperature for sterilization of instruments and appliances can not be too highly commended, but these means have a limited application in dental therapeutics.

Of drugs only such are applicable as can be applied to living structures in sufficient strength or power to destroy the infectious influence without injury, from poisoning or destruction of the tissue involved.

It should be remembered of a germicide under the head of disinfection, that it is not the idea to make repeated applications, but that one application alone should serve to sterilize the wound and prepare it for other less powerful agents which are intended to keep it free from the encroachment of disease producing influences.

This being understood we can readily see that very poisonous drugs in strong solutions may be applied to the limited areas, and to tissues of such limited absorbent capacities, as are generally concerned in operations in connection with the teeth and associated parts. To illustrate, we can safely apply a 1 to 100 solution of the bichloride of mercury, for the purpose of sterilization in the pulp canal, or to an alveolar abscess. But even in such favorable cases much injury could be done by repeated and ontinuous applications for any length of time.

The most useful dental germicides are, bichloride of mercury, carbolic acid, oil of cassia, and peroxide of hydrogen. The bichloride of mercury in solutions of from 1 to 100 or 1 to 500; the diluent being distilled water, or peroxide of hydrogen. This is a powerful poison and should be used with caution. It is not known definitely what the chemical reaction is, but mercuric albuminates are formed (indicating decomposition of the bichloride) and nascent and free chlorine is probably the active agent in the sterilizing process. It may act as a simple poison.

Solutions of carbolic acid in water of sufficient power cannot be made to produce satisfactory germicidal results without using solution of such high concentration that destruction of healthy tissues will follow its use, and most oils with which it will mix readily interfere with its germicidal power. The oil of cassia, however, has been found by Dr. Black to not only maintain but materially increase its range of power. In many cases of dental practice the amount of tissue destroyed by a 95 per cent solution of carbolic acid in water would produce no harmful results, but a solution of such strength is not applicable to pulps or gum tissue where it is important to save all the structure possible. Just what the chemical reaction of carbolic acid and oil of cassia may be we cannot tell, but it is probable that carbolates are formed of the proteid elements and the liberated hydrogen and oxygen uniting with the putrefactive gases, destructive acids are formed which act directly upon the micro-organisms.

The peroxide of hydrogen, the safest of all germicides though not so efficient as those above mentioned, undoubtedly owes its power to the liberation of the extra and loosely held atom of oxygen, with which it readily parts when brought into contact with substances having an affinity for oxygen. In its therapeutic application as a disinfectant it gives up its ogygen to sulphuretted hydrogen $H_2$ S. and ammonia N $H_3$ to form sulphuric acid and nitrous acid gases which attack and destroy the micro-organisms. It is undoubtedly also true that a portion of the oxygen is absorbed by the red corpuscles of the blood and the impaired nutrition of the part reinforced so that the vital resistance of the tissue cells take a prominent part, (by resistance at least) in the sterilizing process.

The disinfecting process be it never so carefully and systematically accomplished, will leave the enfeebled tissues exposed and susceptible to renewed attack, unless guarded and protected by a faithful watch, which will not interfere with the normal healing process. This duty we assign to the antiseptics.

These drugs may be administered internally to be absorbed into the general circulatory system and so produce general and local effects, but as is generally the case in dental practice they may be applied locally with the intent to produce an effect on a limited area.

Drugs capable of producing an alkaline or acid condition in the blood (or cause acid or alkaline secretions of glands) would inhibit the development of bacteria and in this way act as local antiseptics although administered through the general circulatory system.

An antiseptic should be defined and thought of not as a germ killer, but as a germ preventive. To attempt an enumeration of the antiseptic drugs with their method of action and virtues would overreach your time and patience and my ability. But we may refer to some of the most important in connection with the principle involved in their use.

Referring to the published reports of Prof. Koch and other eminent bacteriologists we find that a solution containing one part of bichloride of mercury to 300,000 of water is capable of preventing the growth of micro-organisms, and so on through a long list to a one per cent. (1 to 100) solution of alcohol, until we are inclined to believe that almost anything is good enough to keep out the germ influence. But practically we find these attenuated solutions are of little or no use. These determinations were made in the laboratory where all the environments were fixed and could be recognized and regulated at will. But in the practical application new and diverse circumstances and conditions must be taken into account. If the drug is administered internally we must calculate the effect of the further dilution that the antiseptic will undergo, also the action of the living cells of the organism, the glands and tissues, upon it, to dilute or decompose and render it inert. The same allowances must be made when the drug is applied locally, and in addition the fact that it will be absorbed and dissipated or mechanically removed by the secretions, and so further diluted that these tables can not in any sense be looked to for information as to the amount of any drug that can be efficiently used. The most we can get from them is a basis for clinical experimentation.

We have found by this clinical experimentation that all agents in the form of water or alcohol solution are practically worthless, because quickly absorbed and dissipated. An antiseptic dressing to be efficient must be constantly present and on guard to prevent the encroachment of the enemy, and we are coming to place more dependence on those drugs that are not readily decomposed or dissolved, but which still give up their elements in sufficient quantity under ordinary conditions to make them practicable. Hence we find the essential oils, either alone or as diluents of solids and powders, cerates and wax which will make adhesive mixtures, into which can be incorporated drugs having peculiar antiseptic properties, are coming more and more into use as local applications on the principle of persistent application and gradual absorption. By occasional renewing of these applications they may be kept continuously in service until nature has had a chance to restore the impaired structure to a normal and healthy function. In many conditions the application of these forms of antiseptics are inadmissible because of the difficulty of approach. In such cases the solutions or emulsions may be used by injection or spray, but it is as a rule necessary to make the dressings more frequently, consequently this form is less desirable.

